# Comparison of the osteoinductive potential of preconditioned dental pulp stem cell secretome versus their exosomes

**DOI:** 10.1007/s10266-025-01201-6

**Published:** 2025-11-17

**Authors:** Eman Hany, Ahmed A. Emam, Dina Elzeiny, Ahmed A. Elzeiny, Rana El-Qashty

**Affiliations:** 1 Oral Biology Department, Faculty of Dentistry, Mansoura University, Mansoura, Egypt; 2 Medical Experimental Research Center (MERC), Faculty of Medicine, Mansoura University, Mansoura, Egypt; 3 Medical Biochemistry and Molecular Biology Department, Faculty of Medicine, Mansoura University, Mansoura, Egypt; 4 Medical Biochemistry and Molecular Biology Department, Faculty of Medicine, New Mansoura University, New Mansoura, Egypt; 5 Clinical Pathology Department, Faculty of Medicine, Mansoura University, Mansoura, Egypt

**Keywords:** Mesenchymal stem cells, Conditioning, Secretome, Exosomes, Osteogenesis, Mineralization

## Abstract

This study aimed to compare the effects of osteogenically preconditioned dental pulp stem cells (DPSCs) secretome versus their exosomes on proliferation, migration, osteogenic differentiation, and mineralization of bone marrow stem cells (BMSCs). BMSCs and DPSCs were isolated and characterized. DPSCs were osteogenically preconditioned for 10 days before isolation of their derivatives. The study involved four groups: negative control (BMSCs in complete media), positive control (BMSCs in complete media with osteogenic supplement), secretome group (BMSCs co-cultured with DPSCs-secretome), and exosome group (BMSCs co-cultured with DPSCs-exosomes). BMSCs’ viability through MTT assay and cell migration were evaluated. Mineralization was assessed through Alizarin red S and Von Kossa staining. RT-qPCR was performed for osteogenic gene expression, followed by statistical analysis. The exosome group showed the highest cell proliferation on day 5, but it did not reach statistical significance. Cell migration assay showed more rapid wound closure in the control group. However, after 24 h, the secretome group outperformed the exosome group, while the opposite results were observed after 48 h. The exosome group revealed the highest mineralization and osteogenic marker expressions, followed by the secretome, and finally the control groups, with significant differences between groups. Therefore, osteogenic preconditioning can be considered as an efficient tool for enhancing the osteoinductive influence of DPSCs-derivatives, promoting osteoblastic differentiation and mineralization, and under the tested conditions, exosomes demonstrated greater osteoinductive activity compared to secretome. However, osteogenically committed cells seem to lose their paracrine influence on cell viability and migration.

**Graphical Abstract:**

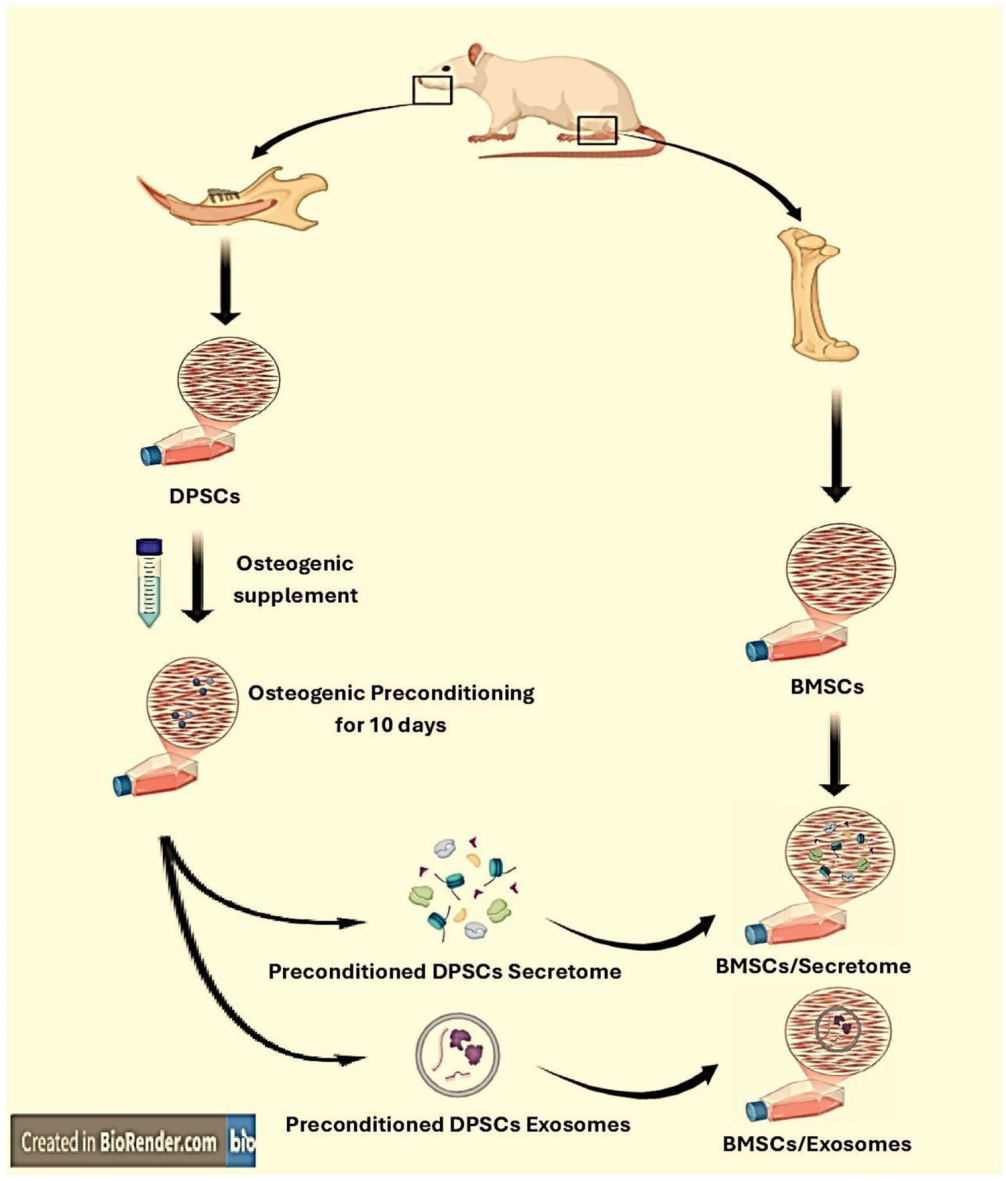

## Introduction

Tissue regeneration research aims to integrate advanced tissue engineering utilizing stem cells, and signaling molecules with biomaterial strategies mediating positive cellular behaviors that promote healing [1]. Over the last decades, various types of stem cells have been applied for bone tissue engineering purposes with very promising results [2]. However, in spite of its evidence-based therapeutic capabilities, stem cell therapy has some limitations, such as the risks of immunogenic rejection, tumor or ectopic tissue formation, and ethical issues [3]. Also, stem cell stability, stemness, heterogeneity, and migration are other challenges [4]. Fortunately, the regenerative effects of stem cells were attributed mainly to their paracrine action [5]. Therefore, cell-free therapy was found to possess similar therapeutic potentials as cell-based therapy, overcoming some of its drawbacks as well as offering better stability, sterilization, and storage for longer times [6].

The secretome is the reservoir of all the cellular products secreted into their extracellular matrix, regulating many important biological functions, such as proliferation, apoptosis, immunomodulation, inflammation, angiogenesis, homeostasis, and adhesion [7]. Dental stem cell-derived secretome has recently gained wide interest in the field of regenerative medicine, showing high therapeutic potential in neurological, cardiopulmonary, and hepatic diseases, as well as bone and dental tissue regeneration [6]. Moreover, dental stem cell secretome exhibits superior properties to non-dental stem cells, particularly in the abundance of transcriptional factors, chemokines, and metabolic-related proteins that enhance cell proliferation, differentiation, and tissue healing [6].

The secretome holds two main components: soluble factors and extracellular vesicles (EVs) [8], where exosomes are small extracellular vesicles (sEVs), less than 200 nm in size, secreted through the endosomal pathway [9]. They consist of a complex mixture of proteins, lipids, and nucleic acids [10], which trigger intracellular cascades that modulate cell behavior upon binding to specific surface receptors on recipient cells [11]. They play a pivotal role in immune regulation, angiogenesis, neuro-regeneration, odontogenesis, and osteogenesis [12].

Preconditioning MSCs before isolating their derivatives can alter their regenerative potential, as the biological nature of secretome or exosomes varies according to the origin and physiological conditions of the parent cells [13]. Due to different microRNA content, derivatives from osteogenically differentiated and non-differentiated MSCs have different osteoinductive capabilities [14].

Despite their broad therapeutic potential, dental stem cell-derived therapies are less explored compared to those from other non-dental cells [15]. Moreover, to our knowledge, a few studies have investigated the osteoinductive capacity of osteogenically preconditioned stem cell-derived- secretome or compared it to that of exosomes. Therefore, this study was conducted to evaluate and compare the effects of osteogenically preconditioned DPSCs-secretome and exosomes on BMSCs.

## Materials and methods

### Ethical statement

All the experimental work was carried out in the Medical Experimental Research Center (MERC), Mansoura University, Mansoura, Egypt. Animal procedures were conducted following the guidelines of the Mansoura University Animal Care and Use Committee (MU-ACUC) with code number MU-ACUC (DENT.R.24.7.10) and according to the Animal Research: Reporting of In Vivo Experiments (ARRIVE) guidelines. Security and safety precautions for laboratory research work were applied according to the recommendations of McCormick-Ell and Connell [16].

### Isolation of mesenchymal stem cells

Ten healthy, male *Sprague–Dawley* rats of 180–200 g weight (4–8 weeks of age) were purchased from Mansoura Experimental Research Center (MERC) to obtain bone marrow and dental pulp tissues. The number of animals was reduced to the least possible number that provided us with enough pulp or bone marrow tissues that yielded enough cell number to perform the experiments while avoiding unnecessary sacrifice of additional animals. Rats were euthanized using an overdose of intraperitoneal sodium thiopental (120 mg/kg; Alfa Chemical Group, Cairo, Egypt).

To obtain rat bone marrow, femoral and tibial bones were dissected and cleaned according to a previous protocol described by El Qashty et al. [17], then transferred to the safety cabinet for end cutting. Bone marrow was then flushed away from the bones using a syringe loaded with culture media supplemented with antibiotics. Finally, the obtained marrow was filtered for removal of any particulates.

Isolation of DPSCs from maxillary and mandibular incisors was performed according to the protocol described by Bertassoli et al. [18], where rats’ skulls were dissected and soft tissue removed, and then, maxillae and mandibles were separated. Each jaw was dissected at the midline into two hemi- jaws which were then cleaned and rubbed meticulously with Betadine, immersed in phosphate-buffered saline (PBS) supplemented with antibiotic, and transferred thereafter to the biological safety cabinet for further manipulation. Under the hood, bone was carefully removed to expose the apical foramen, and a pulp extirpation barbed broach was inserted, watch-windingly rotated, and then withdrawn against the wall of the root canal to obtain intact pulp tissue. The obtained tissues were manually fragmented and then digested using collagenase I (cat. #SCR103, EMD Millipore Corp., Burlington, MA, USA).

The isolated bone marrow and dental pulp were suspended in Dulbecco's Modified Eagle Medium (D-MEM; cat. #L0066-500, BioWest, Nuaillé, France) and then centrifuged. The cell pellets were then incubated in D-MEM supplemented with fetal bovine serum (FBS) (cat. #S1810-500), L-glutamine (cat. #P1012), and penicillin–streptomycin (cat. #100X-L0022), all purchased from BioWest, Nuaillé, France. The media was changed every 3 days. Cell cultures were evaluated daily using an inverted microscope (Olympus, CKX41SF, Tokyo, Japan) till reaching 80% confluence.

### Characterization of mesenchymal stem cells

A BD Accuri C6 cytometer (BD Biosciences, California, USA) and program software were used for flow cytometric immunophenotype determination. Third passage cells were treated with trypsin, followed by washing with PBS and incubation with the following primary antibodies: anti-CD73 purified (cat. #551,123), anti-CD90 PE (cat. #551,401), anti-CD45 FITC MAB (cat. #561,867), all purchased from BD Biosciences, California, USA, and anti-CD34 purified (R&D Systems, cat. #AF6518-SP, Minneapolis, USA). For purified antibodies, fluorescein isothiocyanate fluorophores (FITC, cat. #F143, Thermo Fisher Scientific, Massachusetts, USA) were added to each antibody, followed by their incubation in the dark for 30 min at 4 °C. PBS was then used to rinse the labeled cells, which were then centrifuged for 5 min at 200 × g and resuspended in PBS.

### Osteogenic preconditioning of DPSCs

Osteogenic supplement media was prepared for osteogenic preconditioning of DPSCs for 10 days before the isolation of different cell derivatives (secretome and exosome) following our previous protocol [19] using 100 nM dexamethasone (cat. #D4902), 200 μM L-ascorbic acid 2-phosphate (cat. #A8960), and 10 mM β-glycerol phosphate (cat. #50,020), all purchased from Sigma-Aldrich, Poole, UK.

### DPSCs-secretome preparation

Secretome was prepared from osteogenically preconditioned DPSCs according to our previous protocol [20], where 1 × 10^6^ cells were seeded, and after reaching 80–90% confluence, the DPSCs were washed three times with PBS, and the media was replaced with serum-free DMEM in which cells were incubated for 48 h. The media was then collected, centrifuged at 1200 × g for 5 min, filtered through a 0.2 μm filter to remove cellular debris, and finally stored at -80 °C until use.

### DPSCs-exosomes’ preparation

Exosomes were isolated from cell culture media of osteogenically preconditioned DPSCs using the Total Exosome Isolation Reagent (TEIR) kit (cat. #4,478,359, Invitrogen, Thermo Fisher Scientific, Massachusetts, USA) as instructed by the manufacturer. In brief, cells were cultured in serum-free media for 48 h before exosome isolation, after which the media was collected and centrifuged for 30 min at 2000 g. The supernatant was then incubated with the TEIR kit overnight and finally centrifuged for 1 h at 10,000 g. The exosome pellet was resuspended in PBS thereafter and stored at − 80 °C until usage.

### Characterization of DPSCs’ derivatives

Protein concentrations of DPSCs-secretome and DPSCs-exosome samples were assessed using a bicinchoninic acid Pierce BCA protein quantification assay kit (cat. #23,225, Thermo Fisher, Massachusetts, USA). Isolated DPSCs-exosomes were examined by transmission electron microscopy (TEM) (JEOL 2100 TEM, JEOL Ltd., Tokyo, Japan) to detect the morphology and average particle size, where samples were fixed overnight and mounted on carbon-coated grids for negative staining with 2% phosphotungstic acid for 5 min. After air drying, the samples were observed and photographed.

Immunoflowcytometric analysis was performed to detect the representative protein markers of exosomes: CD63 (FITC Anti-CD63 antibody [AD1], cat. # ab108949) and tumor susceptibility gene 101 (APC Anti-TSG101 antibody [EPR7130(B)], cat. # ab316299), both purchased from abcam, USA.

Particle size and size distribution of DPSCs-exosomes were detected using the Zetasizer Nano ZS90 DLS instrument (Malvern Instruments, Worcestershire, UK). Samples were diluted with PBS at a ratio of 1:20 to decrease particle aggregation and analyzed in triplicates. The polydispersity index (PDI) was analyzed to ensure the homogeneity of the sample. The zeta potential (ZP) of the isolated sample particles was also detected using the same device.

### Study design

#### Negative control

BMSCs were cultured in complete media.

#### Positive control

BMSCs were cultured in complete media with osteogenic differentiation supplement.

#### Secretome group

BMSCs were co-cultured with osteogenically preconditioned DPSCs-secretome (1091.6 μg/mL) with the ratio of 3:7 in each well (secretome:complete media = 3:7) [21].

#### Exosome group

BMSCs were co-cultured with osteogenically preconditioned DPSCs-exosomes at a concentration of 25 μg/mL in each well [22].

### Cell viability assay

Cell viability and proliferation for the negative control, secretome, and exosome groups were assessed on days 1, 3, and 5 using 3-(4,5-dimethylthiazol-2-yl)-2,5-diphenyltetrazolium bromide (MTT) colorimetric assay (cat. #M6494, Thermo Fisher Scientific, Carlsbad, CA). Briefly, culture media was removed, and cells were incubated with MTT reagent (0.5 μg/mL) for 2 h at 37 ℉C. For dissolving formazan crystals, dimethyl sulfoxide (DMSO) was added, and after incubation for 30 min, the absorbance was measured at 550 nm using a microplate reader (Biotek, ELx800, Winooski, VT, USA).

### Cell migration assay

Cell migration was assessed for negative control, secretome, and exosome groups using scratch assay according to the protocol described by Giannakopoulos et al. [23], where BMSCs were grown on coated 24-well plates till a confluent monolayer was visualized. A linear scratch was made with a 100 µl pipette tip, and then, cellular debris was washed away by PBS. Different media, according to each group, were added to the cells and incubated thereafter for 24 h and 48 h at 37°C. Three photographs were captured for each well, and the wound area was measured using ImageJ software (version 2; NIH, Maryland, USA) and compared to the original wounds.

### Alizarin red S staining

Alizarin red S (ARS) staining was used to assess mineralization in the cell matrix after 14 days for all experimental groups as described in a previous study [24]. Briefly, the culture media was discarded, and cells were washed with PBS with Ca/Mg, fixed, and washed again. The cells were then incubated with ARS (cat. #A5533, Sigma-Aldrich, Poole, UK) for 45 min, after which they were washed, and PBS was added. Cultures were observed and photographed with an inverted microscope, and the degree of absorbance was measured by a microplate reader (Biotek, ELx800).

### Von Kossa staining

Von Kossa staining was carried out to detect mineralization for all groups in the cell cultures after 14 days, according to Ball et al. [25]. Briefly, BMSCs were fixed, washed, and then incubated with AgNO_3_ (cat. #209,139, Sigma-Aldrich, Poole, UK) (under UV light), Na_2_CO_3_ (cat. #S263-500, Thermo Fisher Scientific, Carlsbad, CA), and Na_2_S_2_O_3_ (cat. #217,263, Sigma-Aldrich, Poole, UK), respectively, with rinsing twice by dH_2_O in-between each step. Cultures were observed and photographed with an inverted microscope.

### Reverse transcription and real-time quantitative polymerase chain reaction (RT-qPCR)

Total RNA was isolated from BMSCs after 14 days using Direct-zol RNA Miniprep (cat. # R2051, Zymoresearch, CA, USA) supplied with TRI Reagent (cat. # R2050-1-50) according to the manufacturer’s instructions, and then, RNA concentration and purity were measured using the NanoDrop™ 2000/2000c Spectrophotometer (Thermo Scientific, USA). The extracted RNA was reverse transcribed into cDNA using the COSMO cDNA synthesis Kit (cat. #WF10205001, Willowfort, Birmingham, UK) and GeneAmp PCR (Applied Biosystems, USA) under the following conditions: 5 min at 25 °C for primer annealing, 15 min at 45 °C for reverse transcription, followed by 5 min at 85 °C to stop the reaction. The resulting cDNA was amplified using a real-time thermal cycler (Azure Biosystems, USA) with HERA PLUS SYBR® Green qPCR Kit (cat. #WF10308001, Willowfort, Birmingham, UK) under the following thermal cycling conditions: an initial denaturation at 95 °C for 2 min, followed by 40 cycles of 95 °C for 10 s and 60 °C for 30 s.

Expression levels of osteoblast-specific target genes mRNA: collagen 1α1 (*Col1α1*), osteocalcin (*BGLAP*) (*OCN*), osteopontin (*SPP1*) (*OPN*), alkaline phosphatase (*ALP*), and Runt-related transcription factor 2 (*Runx-2*) were detected. Specific primers for these genes were designed using NCBI Primer-BLAST (https://www.ncbi.nlm.nih.gov/tools/primer-blast/) and synthesized by Vivantis Technologies (Selangor Darul Ehsan, Malaysia). Gene expression levels were analyzed using the 2 − ΔΔCt method [26] and normalized to the housekeeping gene Glyceraldehyde-3 phosphate dehydrogenase (GAP-DH). The primer sequences utilized in this study are listed in Table 1.

**Table 1:** Primer sequences for qPCR

Gene	Primers	Sequence
Alkaline phosphatase	Alpl-F Alpl-R	GAC AAG AAG CCC TTC ACA GC ACT GGG CCT GGT AGT TGT TG
Runx-2	Runx2-F Runx2-R	GGA CGA GGC AAG AGT TTC AC GGA CCG TCC ACT GTC ACT TT
Osteocalcin	Bglap-F Bglap-R	GAG GGC AGT AAG GTG GTG AA GTC CGC TAG CTC GTC ACA AT
Osteopontin	Spp1-F Spp1-R	GAT CGA TAG TGC CGA GAA GC ACT CGT GGC TCT GAT GTT CC
Collagen 1α1	Col1α1-F Col1α1-R	CTG GTG AAC AGG GTG TTC CT GGA AAC CTC TCT CGC CTC TT
GAP-DH	Gapdh-F Gapdh-R	TGG GAA GCT GGT CAT CAA C GCA TCA CCC CAT TTG ATG TT

### Digital histomorphometrical image analysis

Quantification of scratch assay and Von Kossa staining surface area were performed using the image processing software Fiji ImageJ (version 2; NIH, Maryland, USA). For the scratch assay, the measuring function was used to measure the width of the scratch at three different points along the scratch for each image. For Von Kossa staining surface area, the Color Deconvolution 2 plugin and Region of Interest (ROI) were used. The staining surface area was measured as a percentage relative to the area of the image. The average staining surface area was calibrated for each well from three different areas.

### Statistical analysis

Results for each group were obtained from three independent experiments; each performed in triplicates. Data were analyzed using GraphPad Prism 9 (GraphPad Software). Quantitative data were described using mean ± standard deviation for normally distributed data after testing normality using the Shapiro–Wilk test. One-way ANOVA, followed by post hoc Tukey’s multiple comparison test, was used to compare the groups for ARS, Von Kossa staining, and RT-qPCR results. The two-way ANOVA test was used to assess the combined effect of two independent factors on a dependent continuous outcome with the post hoc Tukey test for pairwise comparison for MTT and scratch assays’ results. The significance of the results obtained was judged at the (0.05) level.

## Results

### Characterization of mesenchymal stem cells

**Fig. 1:**
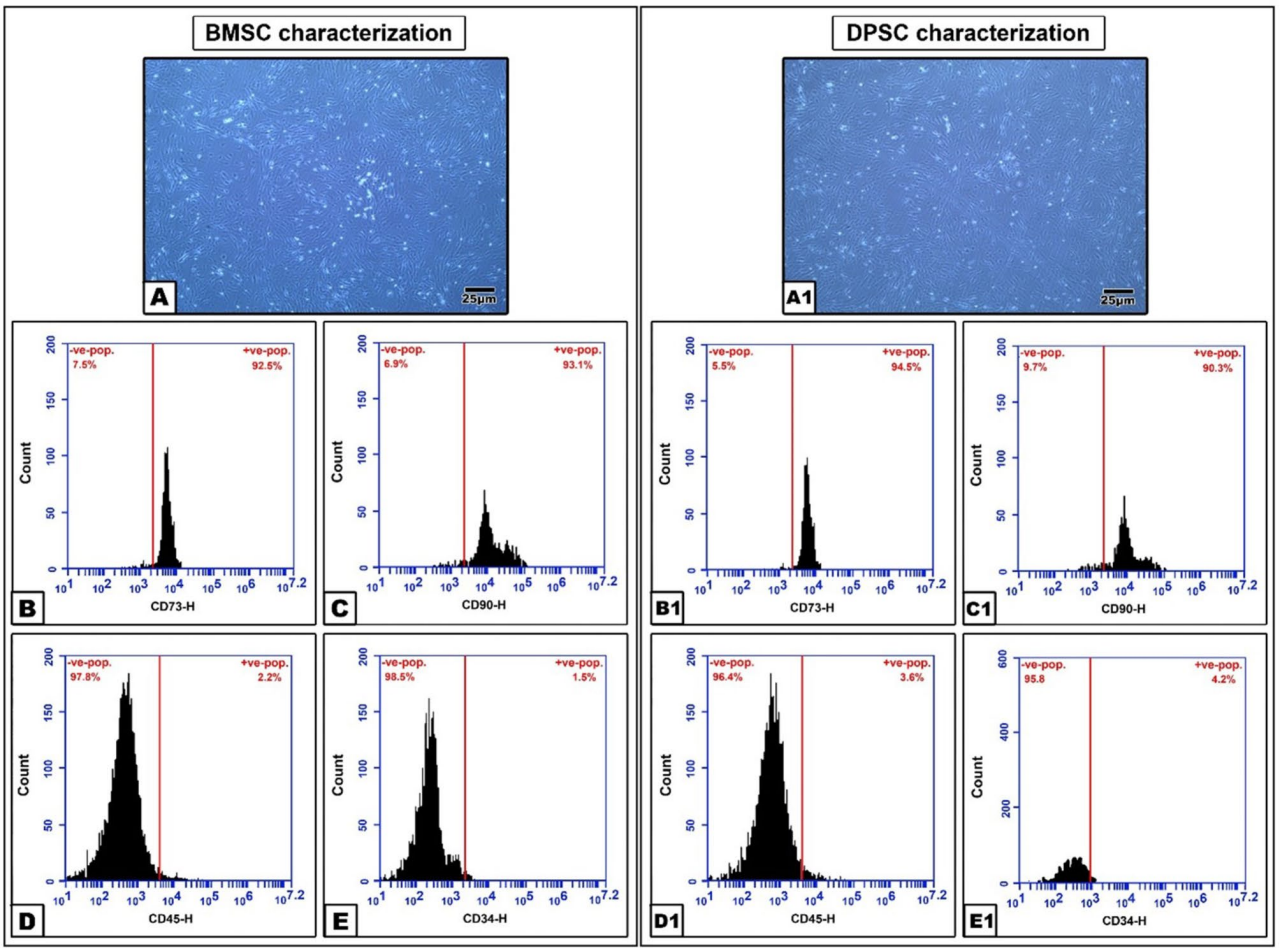
Characterization of isolated MSCs **A**. BMSCs confluence (10 ×) A1. DPSCs confluence (10 ×) **B**–**E** BMSCs flow cytometric histograms B1-E1. DPSCs flow cytometric histograms

Both BMSCs and DPSCs showed typical fibroblast-like, spindle- shaped morphology and adhered to plastic walls (Fig. 1** A, A1**). The cell surface phenotypic marker analyses of third-passage BMSCs and DPSCs were found to be highly positive for the mesenchymal markers CD73 (92.5%, 94.5%) and CD90 (93.1%, 90.3%) while negative for the hematopoietic markers CD45 (2.2%, 3.6%) and CD34 (1.5%, 4.2%), respectively (Fig. 1** B-E, B1-E1).**

### Characterization of DPSCs-exosomes

**Fig. 2:**
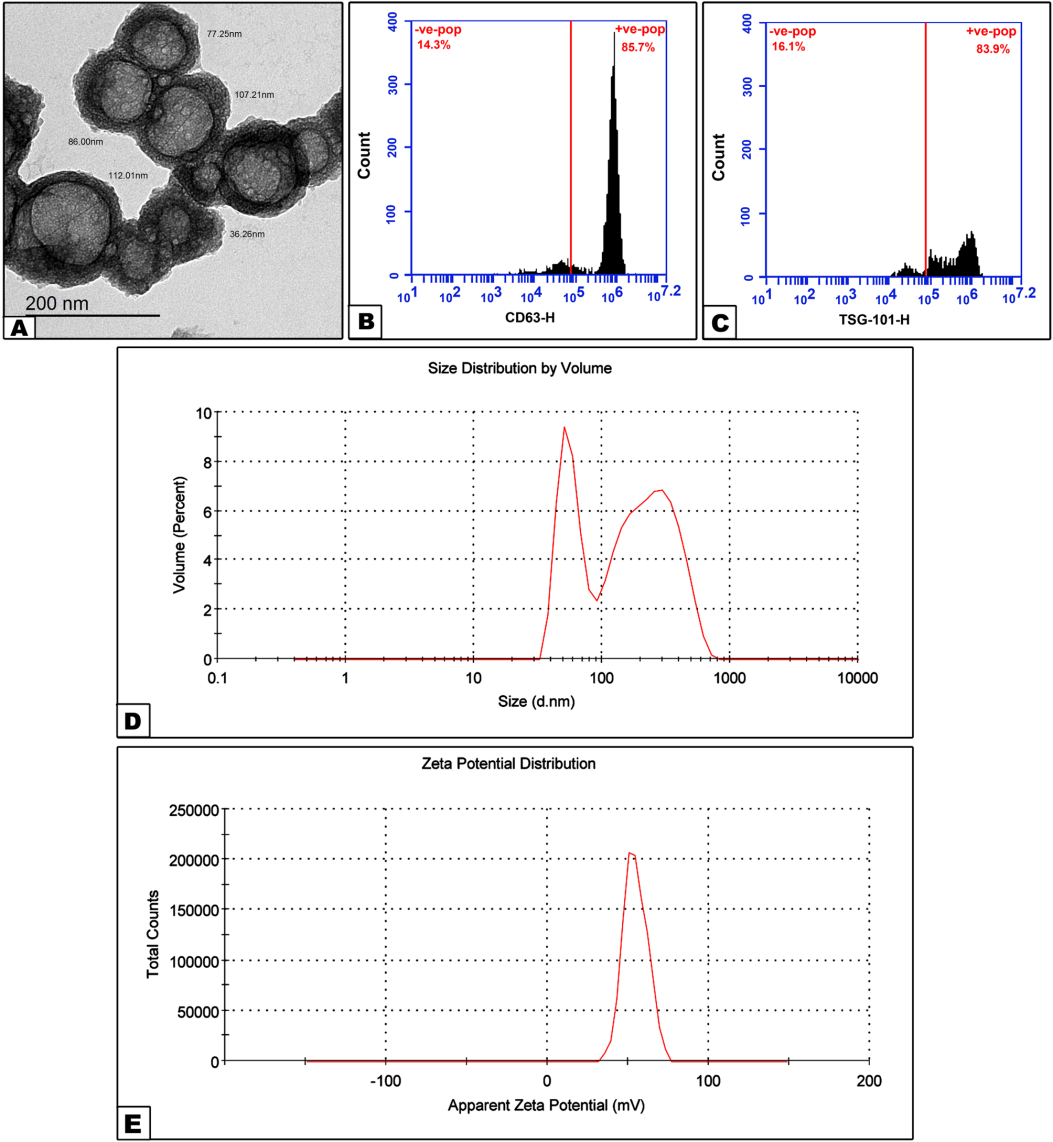
Characterization of DPSCs-exosomes. **A** TEM image, flow cytometric analysis for **B** CD63 and **C** TSG-101 antibodies. **D** DLS particle-size distribution. **E** Zeta potential of DPSCs-exosomes particles

The TEM scanning of DPSCs-exosomes showed typical cup- shaped, clustered, nano-sized vesicles ranging in diameter from 30 to 150 nm and surrounded by bilayer lipid membranes. The DLS zeta sizer analysis results showed the average particle size to be 214.3 nm and ZP to be 54.4 ± 5.2 mV. PDI was found to be 0.172. Flow cytometric analysis of exosomal proteins showed high positive reactions for CD63 (85.7%) and TSG-101 (83.9%), confirming the expression of trans-membranous and cytosolic exosomal markers (Fig. 2).

### Cell viability assay

**Fig. 3:**
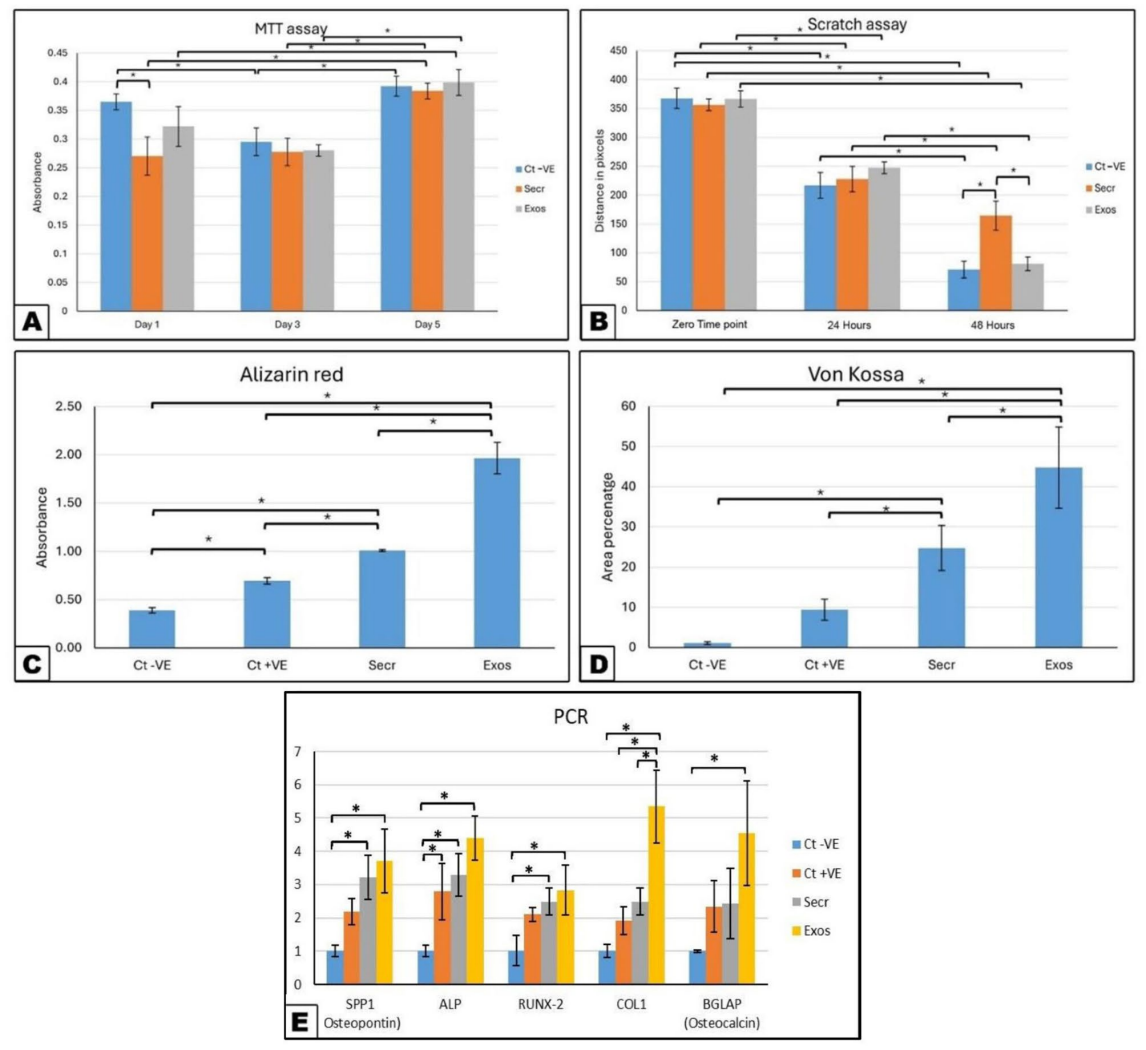
Bar graphs showing statistical analysis results followed by post hoc Tukey test. **A** Two-way ANOVA results of MTT assay. **B** Two-way ANOVA results of scratch assay. **C** One-way ANOVA results of alizarin red S stain D. One-way ANOVA results of von Kossa stain. **E** One-way ANOVA results of RT-qPCR. *Denotes significance

MTT assay was conducted to evaluate the influence of different therapies on cellular viability and proliferation. It showed higher mean values in the control group, followed by the exosome then the secretome groups on days 1 and 3, while on day 5, the exosome group showed the highest value (Fig. 3A, Table 2). Two-way ANOVA analysis revealed a significant interaction between intervention and time factors [F (4, 18) = 3.33, *P* = 0.0330]. Therefore, a simple main effect analysis of the factors was performed.

**Table 2:** Post hoc Tukey test for pairwise comparison of factors affecting MTT assay and scratch cell migration assay

Group time	Negative control	Secretome	Exosome
MTT assay			
Day 1	0.37 ± 0.01	0.27 ± 0.03^a^	0.32 ± 0.03
Day 3	0.30 ± 0.02^*^	0.28 ± 0.02	0.28 ± 0.01
Day 5	0.39 ± 0.02^#^	0.38 ± 0.01^*#^	0.40 ± 0.02^*#^
Scratch cell migration assay			
0	367.68 ± 17.57	356.51 ± 10.35	366.54 ± 14.16
24 h	216.89 ± 22.46^*^	227.70 ± 21.93^*^	247.41 ± 10.28^*^
48 h	70.90 ± 14.45^*#^	164.47 ± 25.24^*#a^	81.11 ± 11.87^*#b^

Data were presented as Mean ± SD, **a**: significance vs control group within the same time point,** b**: significance vs secretome group within the same time point, *: significance vs the first time point within the same group, **#**: significance vs the second time point within the same group at *P* < 0.05

Regarding the simple main effect of the intervention factor, no significance was detected between different groups at different time points, except on day 1, where the secretome group showed a significantly lower value relative to the control (*P* = 0.0021). Therefore, under the conditions of the current study, osteogenic secretome or exosome treatments did not have a significant influence on cell proliferation or viability. Regarding the time factor, the control group showed significantly lower values on day 3 than on day 1 (*P* = 0.0327). Nevertheless, day 5 showed significantly higher viability rates relative to day 3 (*P* = 0.0016). However, in both secretome and exosome groups, cell viability values were significantly higher on day 5 compared to day 1 (*P* = 0.0003, 0.0154) and day 3 (*P* = 0.0006, 0.0002), respectively.

### Cell migration assay

**Fig. 4:**
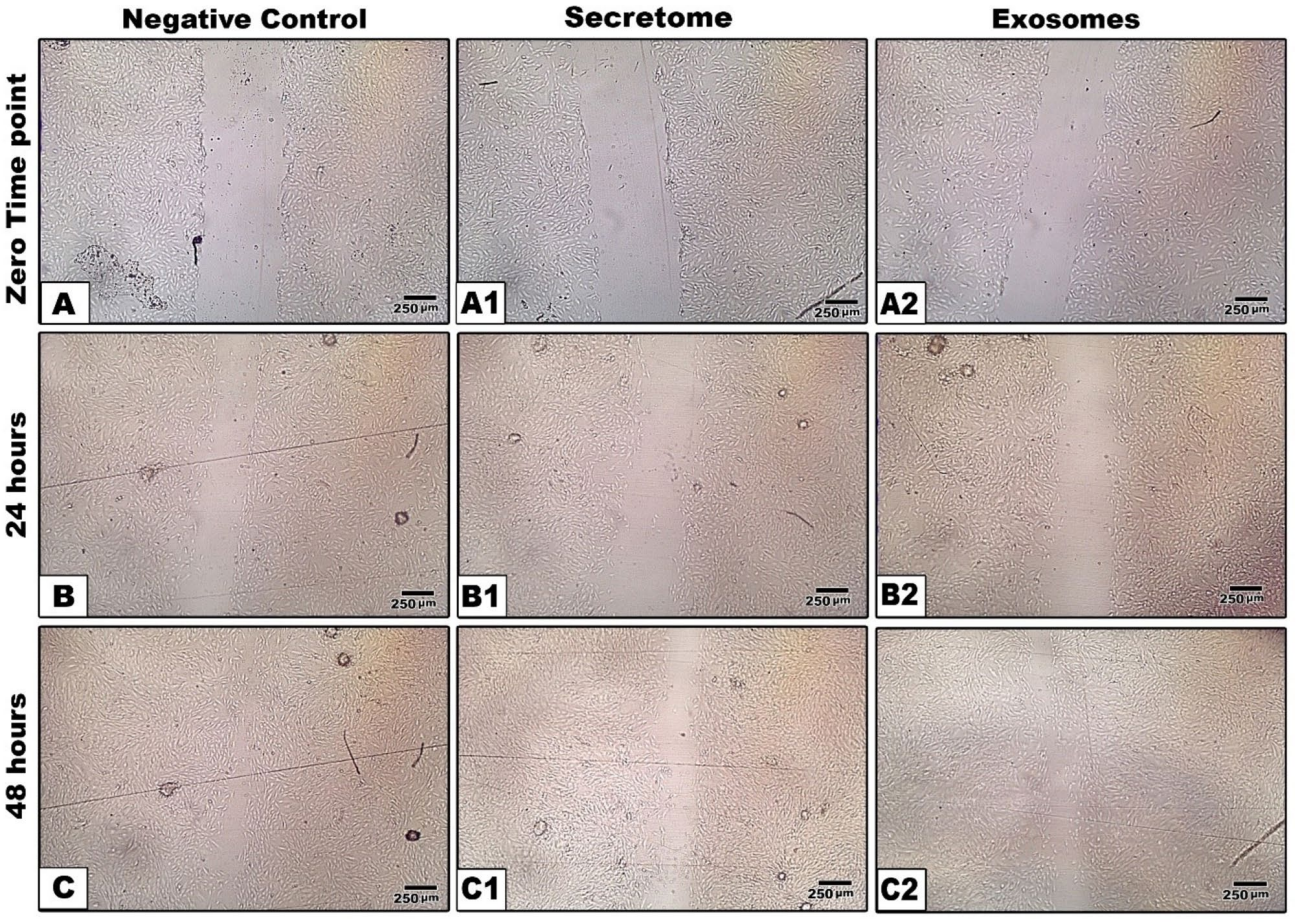
Photomicrographs of scratch assay results of different groups at different time points (10 ×)

A scratch assay was performed to estimate the impact of different therapies on cellular migration where after 24 h, more rapid closure of the midline scratch was observed in the control group, followed by the secretome and then exosome groups. While after 48 h, more closure was observed in the control group followed by the exosome and then secretome groups (Fig. 4). Two-way ANOVA analysis revealed a significant interaction between intervention and time factors [F (4, 36) = 18.23, *P* < 0.0001]. Therefore, a simple main effect analysis of the factors was performed.

As for the simple main effect of the intervention factor, no significant difference was detected between the different groups at 0 and 24 h. However, at 48 h, the secretome group showed significantly larger wound dimensions as compared to the control (*P* < 0.0001), and the exosome groups (*P* < 0.0001). Therefore, osteogenic secretome or exosome treatments did not have a significant influence on cell migration. As for the simple main effect of the time factor, wound dimensions showed a significant reduction in a time- dependent manner through 0, 24, and 48 h for all experimental groups (*P* < 0.0001) (Fig. 3B, Table 2).

### Alizarin red S staining

**Fig. 5:**
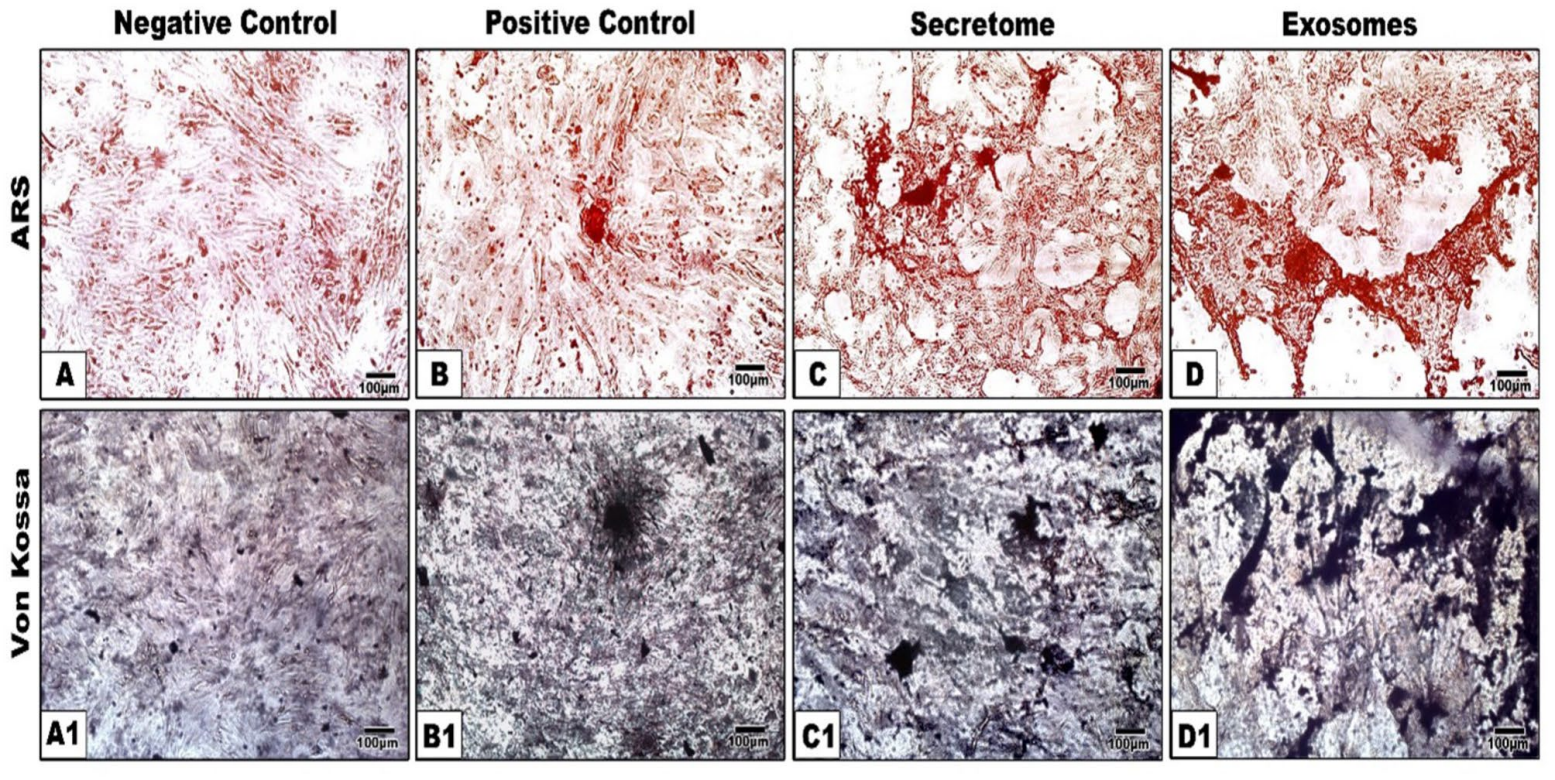
Photomicrographs of Alizarin red S and Von Kossa staining of different groups at different time points (40 ×)

Alizarin red S staining was used to assess the degree of mineralization where calcified nodules were stained red (Fig. 5). The exosome group showed the highest mineral deposit formation, followed by the secretome group, then the positive control group, and finally the negative control group, with a significant difference between all groups (Fig. 3C, Table 3).

**Table 3:** One-way ANOVA analysis followed by post hoc Tukey test for Alizarin Red S and Von Kossa stain results

		Negative control	Positive control	Secretome	Exosome	F	*P* value
ARS	Mean ± SD	0.39 ± 0.03	0.70 ± 0.03	1.01 ± 0.01	1.97 ± 0.16	196.6	< 0.0001*
	Post hoc		*P*1 = 0.0093*	*P*1 < 0.0001* *P*2 = 0.008*	*P*1 < 0.0001* *P*2 < 0.0001* *P*3 < 0.0001*		
Von Kossa	Mean ± SD	1.09 ± 0.34	9.38 ± 2.63	24.76 ± 5.57	44.73 ± 10.09	52.63	< 0.0001*
	Post hoc		*P*1 = 0.1607	*P*1 < 0.0001* *P*2 = 0.0041*	*P*1 < 0.0001* *P*2 < 0.0001* *P*3 = 0.0003*		

Data were expressed as Mean ± SD, *P*: Probability *: significance < 0.05, *P*1: significance vs negative control group, *P*2: significance vs positive control group, *P*3: significance vs secretome group

### Von Kossa staining

Von Kossa staining resulted in the formation of black-stained calcified nodules (Fig. 5) where the exosome group showed the highest calcification concerning area and number of nodules, followed by the secretome group, then the positive control group, while the negative control group showed only minimal mineralization. Statistical analysis revealed significant differences between all groups except between the positive and the negative control groups (Fig. 3D, Table 3).

### RT-qPCR statistical analysis

According to the one-way ANOVA statistical analysis of RT-qPCR osteoblastic gene expression levels, the exosome group showed the highest expression level, followed by the secretome group, then the positive control group, and finally the negative control group (Fig. 3E, Table 4). Both the secretome and exosome groups showed significantly higher values relative to the negative control group for *OPN* (*P* = 0.0111, 0.0033) and *Runx-2* (*P* = 0.024, 0.0082), respectively. For *ALP* gene expression, all groups showed significant differences relative to the negative control group (*P* = 0.0353, 0.0094, 0.0008). For *COL1* gene expression, the exosome group showed significant differences relative to all other groups (*P* = 0.0001, 0.0008, 0.0026). For *OCN* gene expression, only the exosome group showed a significant difference relative to the negative control group (*P* = 0.0118).

**Table 4:** One-way ANOVA analysis followed by post hoc Tukey test for RT-qPCR results

	Negative control	Positive control	Secretome	Exosome	*F*	*P* value
OPN	1.01 ± 0.18	2.19 ± 0.40	3.21 ± 0.67^a^	3.71 ± 0.96^a^	10.93	0.0033*
ALP	1.01 ± 0.17	2.79 ± 0.85^a^	3.30 ± 0.64^a^	4.40 ± 0.66^a^	14.96	0.0012*
Runx-2	1.01 ± 0.45	2.10 ± 0.22	2.49 ± 0.41^a^	2.84 ± 0.76^a^	7.818	0.0092*
COL1	1.01 ± 0.20	1.92 ± 0.46	2.49 ± 0.41	5.35 ± 1.10^abc^	25.93	0.0002*
OCN	1.00 ± 0.03	2.33 ± 0.77	2.42 ± 1.06	4.55 ± 1.57^a^	6.205	0.0175*

Data were expressed as Mean ± SD, *: significance < 0.05, **a:** significance vs negative control group, **b:** significance vs positive control group, **c:** significance vs secretome group at *P* < 0.05

## Discussion

Dental MSCs secretome represents a unique therapy for various pathological conditions [6]. However, increasing evidence has recently been raised that speculates exosomes are the main player in stem cell paracrine-mediated regenerative effects [27]. Moreover, a large body of research has evidenced the benefits of cell preconditioning in promoting their regenerative potentials by activating different signaling pathways [13]. Therefore, the present study was conducted to compare the osteoinductive influence of different osteogenically preconditioned cell-free therapies on BMSCs.

DPSCs were used in this study to obtain different cell-free derivatives, as they are easily accessible, with high proliferation rates and high osteogenic potentials [28]. Secretome and exosomes were derived from osteogenically pre-differentiated DPSCs cultured in an osteogenic supplement for 10 days. In a study by Zhai et al. [14], exosomes were derived from pre-differentiated MSCs after 4, 10, 15, and 20 days of osteogenic induction, and the findings highlighted that exosomes derived after 10 and 15 days expressed the best osteoinductive results. Similarly, another study found that osteogenic exosomes derived within 1–14 days showed a higher osteogenic influence on adipose-derived stem cells (ADSCs) than those derived within 15–28 days of osteogenic induction [29], where exosomal miRNA expression changes during osteogenic differentiation [30].

In the current study, the positive control group showed good mineralization and osteogenic differentiation, where BMSCs are postulated to have an inherent tendency toward osteogenic differentiation owing to their natural source from bone tissues [31]. The secretome group showed higher osteogenic gene expression and mineralization levels than the control groups, where the regenerative impacts of secretome are attributed to its anti-inflammatory, angiogenic, immunomodulatory, and anti-apoptotic effects [32]. In this study, secretome was introduced to BMSC cultures at a concentration of 3:7 secretome to culture media. Zhong et al. [21] tried three different concentrations, 3:7, 5:5, and 7:3, and reported 3:7 as the best concentration regarding osteogenic differentiation. Furthermore, according to Maxson & Burg [33], 25–50% secretome concentration results in a proficient balance between the pH of the medium and the amount of soluble factors.

In accordance with our results, DPSCs-secretome was found to enhance the osteogenic differentiation of MG-63 osteoblast cells [34]. Similarly, Fujio et al. [35] reported that DPSCs-secretome induced human umbilical vein endothelial cells (HUVECs) to express multiple angiogenic factors, as well as enhance wound healing and support bone defect repair in vivo*.* On the other hand, it could not support in vitro mineralization of human fetal osteoblasts. Concomitantly, Sun et al. [36] detected a transitory retarding influence of BMSCs-secretome on osteoblasts regarding proliferation and osteogenic differentiation owing to reduced *Runx-2* expression.

Discrepancies in secretome treatment outcomes are attributed to its diverse composition, which includes factors that may exert positive or negative influence, besides the multiple, different pathways and molecular cues regulating biological processes [37]. Moreover, variable conditions, including collection time, type of parent cells, preconditioning, and concentration, can dramatically modulate the produced secretome components.

DPSC-exosomes were successfully isolated regarding size, morphology, and zeta potential following to the guidelines of the International Society for Extracellular Vesicles MISEV2023 [38]. However, the DLS zeta sizer analysis results showed the average particle size to be 214.3 nm which is larger than the accepted size range for exosomes. This discrepancy can be justified where according to Souza et al. [39], larger size range in DLS analysis could be attributed to the presence of dispersants shifting the particle size to higher values reaching approximately 20% more than TEM results. Moreover, unlike TEM, DLS cannot measure the size of individual particles; otherwise, aggregates may be found, increasing the size measurements tens of times [40]. Additionally, according to the MISEV2023 [38], there is no sharp scientific agreement on the upper and lower size limits for extracellular vesicles, and some isolation methods can produce particles with different overlapping sizes.

The exosome group in this study showed the highest osteoblastic differentiation and mineralization compared to all other groups, in addition to enhanced proliferation on day 5 and improved cell migration after 48 h compared to the secretome group. Regarding the cellular mechanisms underlying their regenerative potential, DPSCs-exosomes were found to regulate inflammatory responses by inhibiting the IL-6/JAK2/STAT3 signaling pathway and promoting M1/M2 macrophage polarization [41]. Moreover, they can enhance cell proliferation and migration besides exerting angiogenic effects [41, 42]. Interestingly, exosomes were suggested to anchor to the extracellular matrix, simulating mineralization matrix vesicles [43].

According to Liu et al. [44], DPSCs-exosomes can modulate osteogenesis by translocating mRNA and miRNA to the recipient cells, where after miRNA analysis of DPSC-exosomes, Hu et al. [45] found that miR-27a-5p in exosomes downregulates LTBP1 (Latent-Transforming Growth Factor Beta-Binding Protein 1), an extracellular matrix protein that plays a key role in regulating growth factors and controls TGFβ1/Smads signaling pathway promoting osteogenesis. In corroboration with the current study results, Wang et al. [46] reported better osteogenic effect of osteogenic DPSCs-EVs than normal DPSCs-EVs attributing this to upregulation of bone morphogenic protein-2 (BMP-2) and ALP, together with increased expression of miR-1246.

Osteogenic exosomes are utilized at variable concentrations. A concentration of 25 µg/ml was applied in this study, where Li et al. [22] compared three commonly used concentrations, 10, 25, and 50 µg/ml, and reported that 25 µg/ml achieved the best osteoinductive results. In line with our results, Xie et al. [47] tested the efficacy of osteogenic DPSCs-exosomes on the differentiation of DPSCs and reported high ALP activity, osteogenic marker expression, mineralization, and upregulated TGFβ1/Smads signaling pathway. Concomitantly, an in vivo trial showed that DPSCs-exosomes enhanced alveolar bone and periodontal regeneration in a periodontitis mouse model [48].

On the other hand, in a study by Wei et al. [49], although migration and mineralization of BMSCs co-cultured with osteogenic BMSCs-exosomes were enhanced, ALP activity and osteogenic marker expression were elevated only in the naïve exosome group, which did not receive osteogenic pre-differentiation*.* The authors postulated that exosomes lose their regenerative potential during osteogenic differentiation of their parent cells.

In the present study, osteogenic DPSCs-derivatives significantly augmented osteogenesis by enhancing multiple osteogenic gene expression promoting mineralization. However, they could not significantly influence cell viability or migration compared to the negative control. This is consistent with the previous studies wherein, in a study by Paschalidis et al. [37], DPSCs-secretome inhibited DPSCs proliferation, which was attributed to the increased pH of the media due to the increased cellular metabolic byproducts. Similarly, in another study, osteogenically induced MSCs showed high osteogenic gene expression but lower proliferation rates, whereas several growth factors in cell secretome showed downregulation after osteogenic induction. The authors postulated that committed cells have compromised paracrine activity after osteogenic differentiation [50]. Also, according to Wei et al. [49], exosome treatment did not affect BMSCs viability.

In the current study, an enhanced regenerative influence was achieved by osteogenic exosomes compared to secretome. These findings were consistent with those of Takeuchi et al. [51], who reported that BMSCs-exosomes outperformed BMSCs-secretome when cultured with BMSCs regarding cell migration, osteogenic and angiogenic differentiation, and achieved better bone regeneration when implanted in a rat calvarial model as well. Also, in a study conducted by Katifelis et al. [52], amniotic fluid-derived MSC-exosomes showed superior results compared to the whole secretome in suppressing inflammation by significantly reducing proinflammatory and increasing the anti-inflammatory markers.

In contrast, in a study by Giannasi et al. [53], ADSCs-secretome showed higher chondroprotective efficiency in an osteoarthritis model than ADSC-exosomes, where secretome significantly augmented tissue inhibitor metalloproteinase 1 and 2 and reduced prostaglandin E2 expression, exhibiting similar particle size and antigen presentation, together with higher vesicular component as compared to exosomes.

Concomitantly, based on the fact that the MSC-secretome comprises EVs and soluble factors, González-Cubero et al. [54] investigated the therapeutic potentials of ADSCs-EVs, soluble factors, or whole secretome and reported the whole secretome to exert a better anti-inflammatory effect than either fraction alone, proving the synergistic mechanism of both fractions. However, in another study, ADSC-exosomes were found to augment neutrophil viability, while their secretome improved their function more, making them both good therapeutic choices [55].

Based on the current findings, DPSCs-derivatives can be considered a potent and safe therapeutic alternative for bone repair. Osteogenic preconditioning can produce cell derivatives with high osteoinductive influence, where osteogenic DPSCs-secretome and exosomes could substantially enhance bone regeneration by promoting osteoblastic differentiation through the upregulation of multiple arrays of growth and transcription factors as well as supporting tissue mineralization, with exosomes presenting higher osteoinductive impact than secretome. However, osteogenically committed cells were found to lose their paracrine influence on cell viability and migration.

Although previous work has shown that in vitro osteogenic differentiation and matrix mineralization for cells under osteogenic induction are reliably detectable by day 14 supporting the use of day 14 as a meaningful time point for confirming osteogenesis [56, 57]; however, this short follow-up period can be considered among the limitations of the present study where longer durations could have further influenced the results. Also, a single concentration of each of secretome and exosomes was tested. Therefore, further studies are required to compare different concentrations of both treatments where concentration-dependent effects are well documented in scientific research. Moreover, subsequent in vivo experimental investigations are required to ensure the safety of the suggested potential therapies.

## Data Availability

All data generated or analyzed during this study are included in this published article.

## Abbreviations

MSCs: Mesenchymal stem cells
BMSCs: Bone marrow-derived mesenchymal stem cells
DPSCs: Dental pulp stem cells
EVs: Extracellular vesicles
MERC: Medical experimental research center
MU-ACUC: Mansoura university animal care and use committee
DMEM: Dulbecco's Modified Eagle Medium
FBS: Fetal bovine serum
FITC: Fluorescein isothiocyanate
PBS: Phosphate buffered saline
TEIR: Total Exosome Isolation Reagent
BCA: Bicinchoninic acid
DLS: Dynamic Light Scattering
PDI: Polydispersity index
DMSO: Dimethyl sulfoxide
ARS: Alizarin red S
RT-qPCR: Reverse transcription and real-time quantitative polymerase chain reaction
